# Serum Myostatin among Excessive Drinkers

**DOI:** 10.3390/ijms24032981

**Published:** 2023-02-03

**Authors:** Candelaria Martín-González, Onán Pérez-Hernández, Alen García-Rodríguez, Pedro Abreu-González, Paula Ortega-Toledo, Camino María Fernández-Rodríguez, Julio César Alvisa-Negrín, Antonio Martínez-Riera, Emilio González-Reimers

**Affiliations:** 1 Departamento de Medicina Interna, Universidad de La Laguna, Servicio de Medicina Interna, Hospital Universitario de Canarias, Tenerife, Canary Islands, 38320 La Laguna, Spain; 2 Departamento de Ciencias Médicas Básicas, Unidad de Fisiología, Universidad de la Laguna, Tenerife, Canary Islands, 38320 La Laguna, Spain

**Keywords:** alcoholism, myostatin, fat mass, sarcopenic obesity

## Abstract

Myostatin acts as a negative regulator of muscle growth. Its effect on fat mass is subject to debate. Among alcoholics, there is a high prevalence of muscle atrophy, and increased fat deposition has been also described in these patients. Myostatin could be involved in these alterations, but its relationships with body composition have been scarcely studied in alcoholic patients. To analyze the behavior of myostatin among alcoholics and its relationship with alcohol intake, liver function, and body composition. We investigated serum myostatin in 59 male patients and 18 controls. Patients were all heavy drinkers admitted with organic complications related to excessive ethanol ingestion. Densitometry analysis was used to assess body composition in 46 patients. Handgrip was assessed in 51 patients. Patients showed lower myostatin values than controls (Z = 3.80; *p* < 0.001). There was a significant relationship between myostatin and fat at the right leg (ρ = 0.32; *p* = 0.028), left leg (ρ = 0.32; *p* = 0.028), trunk (ρ = 0.31, *p* = 0.038), total fat proport ion (ρ = 0.33, *p* = 0.026), and gynecoid fat distribution (ρ = 0.40, *p* = 0.006) but not with lean mass (total lean ρ = 0.07; *p* = 0.63; trunk lean ρ = 0.03; *p* = 0.85; lower limbs ρ = 0.08; *p* = 0.58; upper limbs ρ = 0.04 *p* = 0.82; android ρ = 0.02; *p* = 0.88, or gynoid lean mass ρ = 0.20; *p* = 0.19). In total, 80.43% of patients showed at least one criterion of osteosarcopenic adiposity (OSA). Myostatin was related to OSA obesity. We also observed higher myostatin values among patients with body mass index > 30 kg/m^2^. Serum myostatin was lower among excessive drinkers, and it was related to increased fat deposition among these patients but not to lean mass, handgrip, or bone mineral density.

## 1. Introduction

The terms osteosarcopenic obesity or osteosarcopenic adiposity (OSA) [1] have been coined to define a clinical condition characterized by the progressive loss of muscle mass and function, accompanied by osteoporosis and increased fat deposition. This association is not only an epidemiological one as there is also a complex hormonal and cytokine network involved in the pathogenesis of this syndrome [2]. OSA constitutes a major health problem because its prevalence is very high, especially among the elderly, and each of its three components (sarcopenia, osteoporosis, and altered fat distribution) is associated with frailty and a poor prognosis [3,4].

Age is a major factor involved in the development of OSA, especially osteoporosis and sarcopenia. Some studies reveal that loss of muscle mass begins in the fifth decade at a rate of 0.8% per year [5]. As a consequence, 80-year-old adults have lost approximately 30% of their muscle mass [6]. Some toxic compounds, such as ethanol, are associated with muscle atrophy, so that chronic excessive drinkers may develop a similar degree of sarcopenia at a younger age than that suffered by old nonalcoholic individuals [7]. Indeed, the so-called chronic alcoholic myopathy, characterized by muscle atrophy and weakness, is very frequent among alcoholics, affecting nearly 40–60% of middle-aged heavy drinkers [8], and muscle mass may be reduced by up to 30%. A high proportion of these patients also show osteoporosis and altered body fat distribution [9], although controversy exists regarding the prevalence of obesity among excessive drinkers [10]

Among alcoholics, muscle atrophy may be due to the combined effects of proinflammatory cytokines, peroxidative damage, ethanol-induced increased myostatin expression, and an altered hormonal profile (testosterone, IGF-1, vitamin D, and corticosteroids) [11,12]. Sarcopenia is more marked among alcoholics with cirrhosis [13] because, in this disease, several metabolic changes add to the already commented effects of ethanol on muscle. Liver failure leads to reduced glycogen storage and increased proteolysis. Portal hypertension increases intestinal bacterial translocation and, therefore, leads to the increased secretion of proinflammatory cytokines and oxidative damage. Reduced synthesis of IGF-1 due to liver failure and peripheral conversion of testosterone into estrogens (related to portal hypertension) aggravate muscle atrophy, and hyperammonemia, derived from reduced liver ureagenesis, also strongly contributes to sarcopenia [13] by several mechanisms, including increased myostatin expression [14].

Myostatin is a member of the transforming growth factor β superfamily that is mainly (but not solely) expressed in skeletal muscle acting as a negative regulator of muscle growth but also exerting a negative regulatory role on bone [15]. The role of myostatin on osteosarcopenia in alcoholic patients and/or patients with liver cirrhosis of different causes has been extensively studied, but there is discrepancy in the reported results. For instance, Sato et al. failed to find any relation between myostatin and the skeletal muscle mass index (SMI; assessed by computed tomography) among alcoholics with cirrhosis (in contrast to the negative, significant relationship observed in nonalcoholic patients) [16]. In another study, myostatin levels were lower in cirrhotic patients with sarcopenia [17] and showed a direct (positive) correlation with SMI. Nishikawa et al. [18] did find a positive correlation between ammonia and myostatin and a negative one between psoas muscle index and myostatin in accordance with the pathophysiological mechanisms attributed to ammonia [13]. Therefore, although the physiological effect of myostatin inhibiting muscle growth is undisputed, clinical observations show disparate results in patients with sarcopenia related to liver disease.

In contrast to the well-established inhibitory function of myostatin on muscle growth and differentiation, the effect of this hormone on fat development is controversial [19,20]. There are observations that support that myostatin inhibits adipogenesis in preadipocytes but promotes the transformation of pluripotent stem cells into adipogenesis-committed lineages [20]. Some myostatin gene polymorphisms may be associated with obesity [21], and higher myostatin levels than controls have been reported in obese patients [22]. In obese patients, myostatin was related to insulin resistance, proinflammatory cytokines, and the presence of three or more criteria of the metabolic syndrome [23]. Therefore, myostatin is likely involved in sarcopenic obesity [24,25,26], but its role in alcoholic patients with OSA is a matter open to debate, especially considering the discrepancy that exists in relation to the true prevalence of adiposity among excessive drinkers.

Based on these facts, the aim of the present study is to analyze the role of myostatin among alcoholics and its relationship with alcohol intake, liver function, and body composition.

## 2. Results

**Figure 1:**
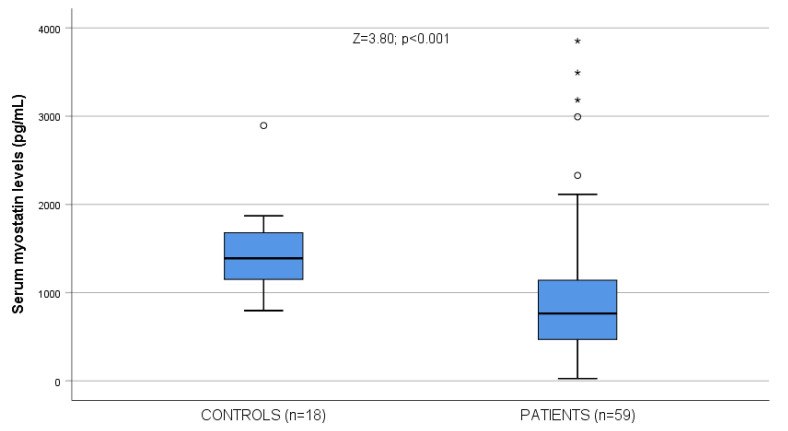
Serum myostatin levels in patients (n = 59) and controls (n = 18). Circles correspond to outliers, and asterisks correspond to extreme outliers.

Patients showed lower myostatin values (978.48 ± 805.84 pg/mL) than controls (1440.89 ± 479.62 pg/mL, Z = 3.80; *p* < 0.001, Figure 1), although patients with cirrhosis showed a nonsignificant trend to higher values (996.10 ± 810.13 pg/mL) than patients without cirrhosis (963.61 ± 814.86 pg/mL; Z = 0.24; *p* > 0.20; NS). Some biological differences among patients with and without cirrhosis and among patients and controls are shown in Table 1.

### 2.1. Ethanol Ingestion

Patients had been heavy drinkers (median = 160; interquartile range (IQR) = 96–240 g ethanol/day) during many years (median = 34; IQR = 20–43 years). Among patients, myostatin was not related to the daily ethanol ingestion (ρ = 0.05; *p* = 0.70) or the duration of the drinking habit (ρ = 0.02, *p* = 0.67) and was also not related to age (ρ = 0.07; *p* = 0.60). We failed to find any relationship between serum GGT and myostatin (ρ = −0.19; *p* = 0.14) or MCV and myostatin (ρ = −0.22; *p* = 0.09).

### 2.2. Liver Function

No relationships were observed when myostatin levels were compared with prothrombin (ρ = 0.05; *p* = 0.70), bilirubin (ρ = 0.03; *p* = 0.81), or albumin (ρ = 0.01; *p* = 0.96). Although Child’s B and C patients showed a trend to higher myostatin levels, these were not significantly different of those observed among Child’s A patients (KW = 2.83; *p* = 0.24). Only six patients with cirrhosis developed encephalopathy, and 10 patients showed ascites, but no differences were observed when myostatin was compared among patients with or without these features (Z = 0.68; *p* = 0.50 and Z = 1.78, *p* = 0.08, respectively).

### 2.3. Body Composition

**Figure 2:**
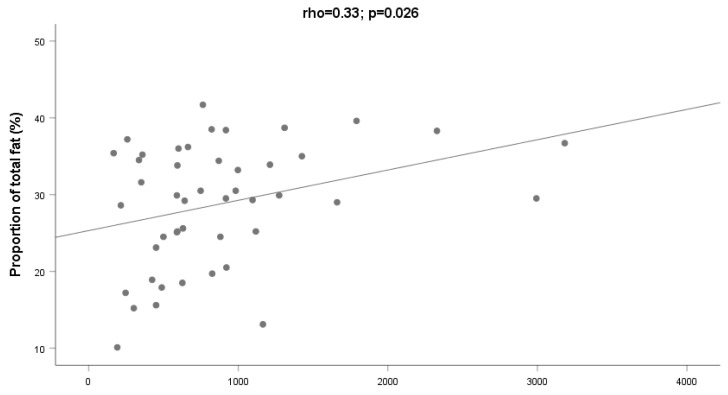
Relationship between serum myostatin and proportion of total fat among heavy drinkers (N = 46).

We found significant relationships between myostatin and the proportion of total fat (ρ = 0.33, *p* = 0.026, Figure 2) and gynoid fat distribution (ρ = 0.40, *p* = 0.006). We also found significant relationships between myostatin and the absolute amount of fat at the right leg (ρ = 0.32; *p* = 0.029), left leg (ρ = 0.32; *p* = 0.028), and trunk (ρ = 0.31; *p* = 0.038). In all these cases, the relationships between myostatin and fat variables were independent of age or the presence or not of cirrhosis, as assessed by stepwise multiple regression analyses (Table 2).

Myostatin was related to fat at lower limbs (ρ = 0.34; *p* = 0.023) but not at upper limbs (ρ = 0.27; *p* = 0.07). Additionally, a significant relationship was observed between myostatin and the total fat/total lean index (ρ = 0.29; *p* = 0.049).

**Figure 3:**
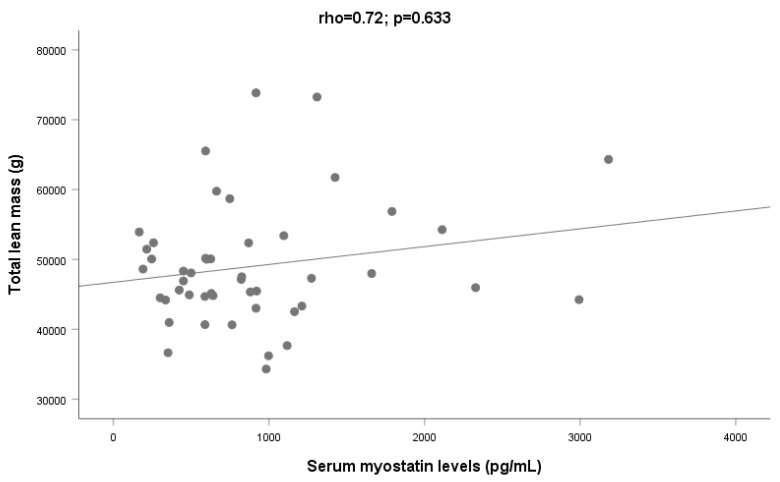
Relationship between serum myostatin and total lean mass among patients (N = 46).

On the other hand, no relationships were observed between myostatin and variables related to lean mass (total lean ρ = 0.072; *p* = 0.63 (Figure 3); trunk lean ρ = 0.03; *p* = 0.85; lower limbs ρ = 0.08; *p* = 0.58; upper limbs ρ = 0.04 *p* = 0.82; android ρ = 0.02; *p* = 0.88, or gynoid lean mass ρ = 0.20; *p* = 0.19).

There was no relationship between myostatin and handgrip (ρ = 0.13; *p* = 0.42), but there was a strong inverse correlation between age and handgrip (ρ = −0.51; *p* < 0.001) and a direct one between handgrip and BMI (ρ = 0.30; *p* = 0.04).

**Figure 4:**
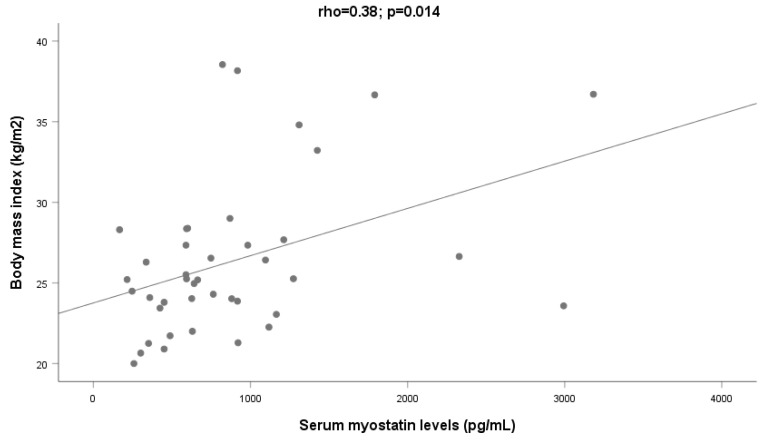
Relationship between serum myostatin and body mass index among patients (N = 47).

A significant relationship was observed between myostatin and BMI (ρ = 0.38, *p* = 0.014; Figure 4) that was even stronger when only patients without ascites (n = 38) were considered (ρ = 0.42; *p* = 0.010).

**Figure 5:**
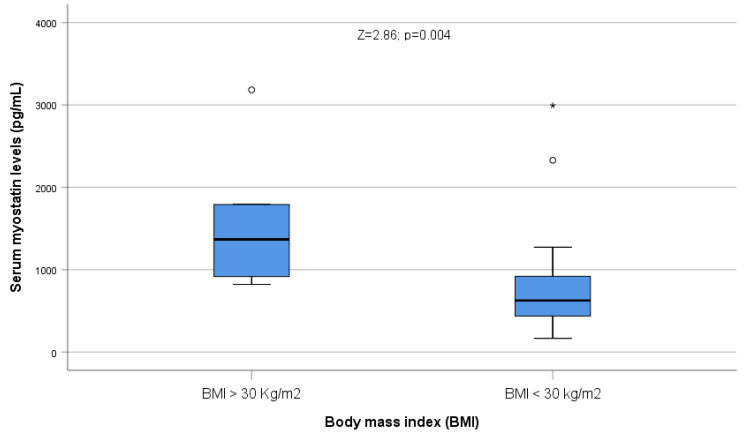
Serum myostatin levels were significantly higher among patients classified as obese according to BMI. Circles correspond to outliers, and asterisks correspond to extreme outliers.

Serum myostatin levels were significantly higher among patients classified as obese according to BMI (>30 kg/m^2^; Z = 2.86; *p* = 0.004, Figure 5).

A stepwise logistic regression analysis comparing obesity with myostatin, age (both as dichotomic variables according to medians), and cirrhosis showed that myostatin was the only variable independently related to obesity, and the same happened when a multiple correlation analysis was performed between BMI as the dependent variable and myostatin, age, and the presence of cirrhosis (Table 3).

No relationships at all were observed when myostatin was compared with bone variables (for instance, femoral neck T-score (ρ = 0.07; *p* = 0.64), total hip T-score (ρ = 0.16; *p* = 0.30) lumbar spine T-score (ρ = 0.10; *p* = 0.52), total bone mineral content (ρ = 0.24; *p* = 0.12), or bone mineral density (ρ = 0.26; *p* = 0.08)).

Regarding the OSA criteria, forty-two patients (77.78%) fulfilled the criterion of sarcopenia according to handgrip strength and 56.76% showed reduced lean appendicular muscle mass. Thirty-four (70.83%) patients were classified as obese according to the proportion of total fat; 22 (45.83%) had osteopenia and only 5 (10.41%) had osteoporosis. Twenty-four patients showed an android to gynoid fat ratio < 1. As expected, age was related to OSA obesity (T = 2.85; *p* = 0.007), OSA handgrip (T = 3.36; *p* = 0.002), and OSA lean mass (T = 2.38; *p* = 0.023). Age was significantly higher among those with at least one OSA criterion (T = 3.44; *p* < 0.001), and among those with three or more OSA criteria (T = 3.65; *p* < 0.001). No relationships were observed among the presence of cirrhosis and OSA criteria.

**Figure 6:**
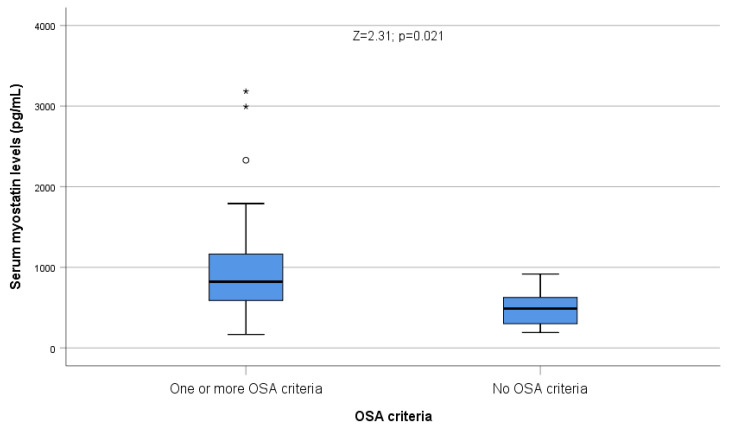
Serum myostatin levels were significantly higher in patients with at least 1 OSA criterion (80.43%). Circles correspond to outliers, and asterisks correspond to extreme outliers.

Serum myostatin was significantly higher among patients with at least one OSA criterion (80.43%) than among the remaining patients (Z = 2.31; *p* = 0.021, Figure 6), but we failed to find differences in myostatin among patients with three or more OSA criteria and the remaining ones (Z = 1.28; NS).

Analyzing separately the relationships between myostatin and OSA criteria, the only significant association was detected between serum myostatin and obesity (proportion of total fat > 25%): the proportion of fat was significantly lower among patients with myostatin levels below the median (Z = 2.15; *p* = 0.037), but myostatin was not related to any other criterion of OSA (Table 4).

However, when performing a logistic regression analysis comparing OSA fat with age, myostatin (both as dichotomic variables according to median values) and cirrhosis, myostatin was displaced by age, which remained as the sole variable related to OSA fat.

Among patients with obesity according to the OSA criteria, no differences were observed in myostatin values between patients with hypertension or diabetes when compared with patients without hypertension (T = 0.94; *p* = 0.36) or diabetes (T = 0.23; *p* = 0.82), and no significant relationships were observed between myostatin and cholesterol (ρ = 0.07, *p* = 0.70), triglycerides (ρ = 0.12, *p* = 0.53) or the index HDL/LDL cholesterol (ρ = 0.14; *p* = 0.45). We also failed to find any statistical relationship between myostatin and these variables when all the patients were included.

## 3. Discussion

This study was performed to evaluate the role of serum myostatin among alcoholic patients, especially analyzing the relationship between myostatin and body composition.

Myostatin serum levels of the patients included in this study were lower than those reported in previous studies. Muscle is the main organ involved in myostatin synthesis [27]. Therefore, the finding of lower myostatin levels in alcoholic patients might be related to the fact that appendicular skeletal muscle mass was markedly reduced in nearly 57% of the patients (following OSA criteria), a prevalence in accordance with the well described chronic myopathy of heavy drinkers.

As previously commented, many factors other than myostatin may contribute to sarcopenia in alcoholics. Reduced muscle mass leads to an absolute reduction of muscle myostatin secretion, partially explaining the lower values of myostatin observed among patients and the nonsignificant trend to a direct relationship (higher myostatin among patients with more strength) observed between myostatin and handgrip strength (Table 4). This is in apparent opposition with the function of myostatin as a negative regulator of muscle mass [28]. A direct relationship between myostatin and muscle mass has also been reported by other authors, as recently reviewed [26].

Heavy alcohol consumption is associated with the increased secretion of proinflammatory cytokines that may promote muscle atrophy [29] and may modulate myostatin effects; both stimulatory [30] and inhibitory [31] actions having been reported. The secretion of proinflammatory cytokines is increased in patients with cirrhosis. In addition, hyperammonemia (a factor able to increase myostatin secretion [14]) is related to liver dysfunction. Therefore, it is not surprising that myostatin levels in alcoholics with cirrhosis may differ from those observed in alcoholics without cirrhosis. Tsien et al. reported increased myostatin expression in patients with cirrhosis [32], in accordance with data previously obtained by García et al., who found a fourfold increase in serum myostatin in 36 patients with cirrhosis compared with 6 controls [33] and also with the results of an experimental study in which myostatin seemed to activate several pathways involved in liver fibrous tissue deposition [34]. In a larger study, including a total of 198 patients with cirrhosis (125 nonalcoholic), Nishikawa et al. [18] reported higher myostatin values among Child B or C patients. These authors also found that higher myostatin levels were associated with higher mortality. Myostatin was related in that study with muscle mass loss and hyperammonemia. By contrast, we found that myostatin levels were lower among patients, despite the severe liver failure observed in some of the included patients with cirrhosis, six of whom developed chronic encephalopathy. However, a slight, nonsignificant trend to higher values of myostatin was observed in patients with cirrhosis compared with patients without cirrhosis, but no relationship was detected between myostatin and liver function impairment.

In contrast, we observed a clear-cut relationship among myostatin and fat mass. The direct relationship of myostatin and fat mass (that was independent of age or cirrhosis) was observed when myostatin was compared with crude data of fat mass at the right leg, left leg, and trunk; when myostatin was compared with fat at lower limbs; when myostatin was compared with the obesity criterion according to BMI and also when it was compared with the OSA criterion for obesity (although in this case the relation of OSA with age was more marked); and with the ratio total fat mass/total lean mass. Therefore, all these data support that, in this study, a direct relationship does exist among myostatin and fat mass. This finding is in accordance with results reported by other authors, such as Amor et al. [22], who found higher serum levels of myostatin in severely obese patients compared with controls and with the observations recorded in several experimental studies strongly suggesting that myostatin may promote adipogenesis. In this sense, Lin et al. reported decreased adipogenesis in myostatin knockout mice [35]. Other studies showed that myostatin promoted the adipogenic differentiation of stem cells [36,37], but Lei et al. report an inhibition of adipogenesis by myostatin [38].

The relationship of myostatin with fat tissue is complex. In experimental studies, inhibition of myostatin led to the transformation of white adipose tissue into brown adipose tissue and provoked an increase in fatty acid oxidation and energy expenditure [39]. Strikingly, the effects of myostatin on fat and lean mass were observed when myostatin gene expression was inhibited in muscle, but no effect was observed when the inhibition took place in adipose tissue [40]. Possibly, the effects provoked by the inhibition of myostatin in muscle leading to increased fatty acid oxidation and reducing fat mass provide the fuel necessary to meet the increased metabolic demand of hypertrophied muscles.

Some data support an indirect effect of myostatin on muscle and fat mass. Myostatin would attenuate the effects of growth hormone on the liver [41]. Growth hormone (GH) is heavily involved in lipid and fat metabolism [42]. Therefore, it could be speculated that attenuation of GH’s action by myostatin could be associated with increased fat deposition.

The effects of cytokines on myostatin secretion have been also explored. In a recent experimental study, Li et al. provide data that suggests that myostatin-mediated muscle wasting observed in experimentally induced lung carcinoma is governed by TNF-α, pointing to the possible existence of a TNF-α/myostatin axis [43]. There is general agreement that adipose tissue—especially trunk fat—induces TNF-α secretion [44], and it is also clear that proinflammatory cytokines induce muscle wasting and loss of adipose tissue [44,45]. Carvalho et al. found a positive correlation between TNF-α and myostatin in obese patients, reporting increased myostatin levels in obese patients with at least three features of the metabolic syndrome (“unhealthy phenotype”) [23]. These results are not supported by this study. We found that myostatin was directly related to trunk fat and gynoid fat accumulation, which is considered a “protective” fat. In addition, we failed to find any relation among myostatin and diabetes, hypertension, or dyslipidemia.

What is the relevance of the direct relationship observed between myostatin and fat in alcoholics? Fat accumulation in these patients has a complex pathophysiology, influenced by many factors not only related to metabolic disturbance but also to lifestyle, nutrition, and even social status [10]. Further studies are required to disentangle the connections between the proinflammatory state of alcoholics, especially if cirrhosis ensues, the altered fat deposition and muscle atrophy of these patients, and the altered myostatin levels. Although the present study should be viewed as a preliminary report due to the small number of patients, the results here suggest the possibility of a cross talk between myostatin and fat mass in alcoholic patients. Many alcoholics with chronic myopathy are severely undernourished. It could be hypothesized that sarcopenia, through decreased myostatin secretion, would “protect” the alcoholic patient from obesity, although many factors other than myostatin are involved in the altered nutritional status in these patients.

As with fat mass, relationships between myostatin and bone are controversial. For instance, Ma et al. found no relationship between BMD and myostatin in healthy postmenopausal women [46], a result in accordance with the data here reported. However, other researchers have reported inverse correlations between myostatin and BMD [47,48]. Available experimental data indicate that myostatin knock-out mice show increased BMD that was attributed to increased weight (the effect disappeared with unloading [49]), and not to a direct effect of myostatin on bone cells. However, later it was suggested that myostatin could inhibit the recruitment and differentiation of osteoprogenitor cells [50,51]. Myostatin may also activate osteoclasts [52]. The relevance of these mechanisms in alcohol-mediated bone changes is unknown.

This study has several limitations. The sample is relatively small. A lack of relationship (or difference) between two variables in a small sample could be attributed to a type II error. This could be the case regarding the lack of relationships among myostatin and handgrip or among myostatin and BMD or BMC, but this is unlikely regarding other variables related to lean mass or bone mass since the lack of a relationship was absolute without any trend that could suggest an effect of the sample size.

The fact that only men were included in this study may constitute another limitation of this research; however, on the other hand, although OSA criteria include different cut-off points for lean appendicular mass, handgrip strength, and obesity for men and women, relationships between myostatin and the crude DEXA-assessed simple variables could be distorted if men and women would have been analyzed together. As shown in Table 4, a high proportion of patients show some OSA features. It has been reported that myostatin is involved in the development of OSA [24,25] and probably also in alcoholics, but in this study, in accordance with the results obtained analyzing the simple variables derived from DEXA, the only OSA criterion associated with myostatin alteration was obesity. Although the relationship was displaced by age when a multivariate analysis was performed, this last result also underscores the importance of myostatin in the adiposity of alcoholic patients.

## 4. Materials and Methods

We prospectively included 59 patients aged 59.25 ± 11.09 years, consecutively admitted to our hospitalization unit due to organic complications related to excessive ethanol ingestion. Only men were included given the marked differences in body composition among men and women and the possibly different relationship between myostatin and muscle mass among men and women [53].

Patients who consumed other drugs besides tobacco were not included in the study. Alcohol consumption was assessed by direct inquiry (to the patients and/or their relatives), recording the type of beverage and the daily amount consumed was estimated using the following formula: volume of ethanol consumed (in g) = degree of beverage (in %) × beverage volume) × alcohol density (0.8). The control group consisted of 18 healthy male hospital workers, aged 54.44 ± 8.10 years (T = 1.70; *p* = 0.093, NS) and were drinkers of less than 10 g ethanol/day. Individuals included in this control group also underwent complete laboratory evaluation, assessment of BMI, and handgrip strength (Table 1).

In addition, to complete routine laboratory evaluation, patients also underwent abdominal ultrasound (US) examination. The presence of splenomegaly and/or portal dilatation and a heterogeneous liver structure and irregular shape, together with altered levels either of albumin (<3.5 g/dL), bilirubin (>2.0 mg/dL), or prothrombin activity (<60%), are used to classify the patients having cirrhosis, a condition fulfilled by 27 out of the 59 patients, whereas the remaining 32 were classified as patients without cirrhosis. To achieve a global assessment of liver function, we applied the Child–Pugh score [54,55] to the whole sample, despite being aware that this score was initially designed as a prognostic tool only for patients with cirrhosis. The Child score is based on the alteration of the following variables: serum albumin, bilirubin, prothrombin activity, and presence/severity of ascites and/or encephalopathy (Table 5).

### 4.1. Laboratory Assessment: Myostatin

Within the first 72 h after admission, blood samples were taken at 8.00 a.m. in fasting conditions and were immediately frozen at −20 °C. Serum myostatin levels were determined by quantitative sandwich enzyme immunoassay technique (R&D Systems, Abingdon Science Park, Abingdon, UK). Each serum sample needed a pretreatment with acid hydrolysis and posterior adjustment to PH 7.4 with HEPES buffer to cleave the active peptide (myostatin) of the original propeptide. We have strictly followed the recommendations of the ELISA manufacturer (R&D Systems, Abingdon Science Park, Abingdon, UK). The yellow final complex was measured at 450 nm in a microplate spectrophotometer reader (Spectra MAX-190, Molecular Devices, Sunnyvale, CA 94089, USA). The calibration curve was prepared with authentic myostatin standards. The coefficient of correlation of the standard curve was 0.997. The detection limit of this assay was established at 2.25 pg/mL; the intra and interassay CV were 3.23% and 4.23%, respectively. The antibody used to coat wells of the plate in this ELISA kit is a human specific monoclonal one. This assay recognizes natural and recombinant mature myostatin. No significant cross-reactivity or interference was observed with other peptides as activin RIIA, activin RIIB, decorin, GASP-2, GDF-11, and GDF-15. The molecules listed previously were prepared at 50 ng/mL in calibrator diluent and assayed for cross-reactivity.

Routine laboratory testing was done for all patients and controls. We recorded prothrombin activity, bilirubin, and albumin, and further calculated the Child–Pugh score. Mean corpuscular volume (MCV) and gamma glutamyl transferase (GGT) were considered “markers” of ethanol consumption.

### 4.2. Body Composition

Forty-six patients underwent a total body composition analysis, recording fat and lean mass at right arm, left arm, right leg, left leg, trunk, and total body, as well as gynoid and android fat and lean mass. This was assessed by dual-energy X-ray absorptiometry with a LUNAR PRODIGY ADVANCE device, General Electric, Piscataway, NJ, USA), following standard criteria [56]. We also recorded body mass index (as weight (in kg)/height (in m^2^)). Handgrip (dominant hand) was also assessed by a Collins dynamometer in 51 patients. Impaired clinical conditions impeded assessment of handgrip in the remaining 8 patients and also impeded DEXA assessment in 13 patients and recording of height/or weight in 12 patients.

In order to assess the presence or not of osteosarcopenic obesity (OSA), we followed recently reported criteria [57,58], defining sarcopenia as a handgrip strength lower than 28 kg (OSA handgrip) and an appendicular skeletal mass index (appendicular skeletal mass (in kg)/height (in m^2^)) lower than 7.26 kg/m^2^ (OSA lean mass); OSA osteoporosis as a femoral neck T-score ≤ −2.5; osteopenia as a femoral T-score ≤ 1; and OSA obesity as percent body fat ≥ 25%. We also calculated OSA android/gynoid fat distribution ≤ 1 (as a surrogate of visceral fat).

### 4.3. Statistics

The Kolmogorov–Smirnov test was used to assess if the variables to be analyzed were normally distributed or not. Nonparametric tests, such as Mann–Whitney´s U test and Kruskal–Wallis test and Spearman´s correlation analysis were used to analyze differences or correlations among nonparametric variables. When the variables subjected to analysis showed a normal distribution, Student´s *t* test, variance analysis, and Pearson´s correlation analysis were used. We also performed multivariate analyses (stepwise logistic regression and/or multiple correlation analyses) in order to test the independence or not of the relationships between myostatin and fat variables. All these analyses were performed with the SPSS program version 25 (Chicago, Ill., USA).

The study protocol was approved by the local ethical committee of our Hospital (number 2017/50) and conforms to the ethical guidelines of the 1975 Declaration of Helsinki. All the patients gave their written informed consent.

## 5. Conclusions

We conclude that serum myostatin was lower among excessive drinkers, among whom sarcopenia was observed in 57% of cases and reduced handgrip strength in 77% of cases. Myostatin levels were independently related to increased fat deposition among alcoholics (observed in 71% of patients) but not to bone mass, lean mass, or handgrip strength. Obese alcoholic patients showed higher myostatin levels, a result that does not depend on the presence of liver cirrhosis or age.

## Figures and Tables

**Table 1:** Some biological differences among patients and controls and among patients with and without cirrhosis.

	Patients (n = 59)	Controls (n = 18)	Z(T); *p*	Cirrhosis (n = 27)	No Cirrhosis (n = 32)	Z(T); *p*
Age (years)	59.25 ± 11.09	54.44 ± 8.10	T = 1.70; 0.093	59.19 ± 9.48	59.31 ± 12.43	T = 0.44; NS
Myostatin levels (pg/mL)	978.48 ± 805.84 763.08 (450.36–1164.48)	1440.89 ± 479.62 1389.22 (1147.08–1694.79)	Z = 3.80; *p* < 0.001	996.10 ± 810.13 763.08 (450.36–1273.24)	963.61 ± 814.86 786.94 (459.79–1102.74)	Z = 0.24; NS
Handgrip (kp)	17.20 ± 14.07	38.88 ± 9.73	T = 5.89; *p* < 0.001	16.04 ± 10.99	18.22 ± 16.482	T = 0.56; NS
BMI (kg/m^2^)	26.66 ± 5.16 25.26 (23.80–27.68)	25.34 ± 2.28 24.41 (23.72–26.37)	Z = 0.54; *p* = 0.59	26.44 ± 4.74 25.26 (23.71–26.51)	26.90 ± 5.66 26.06 (23.80–28.30)	Z = 0.27; NS
Ethanol ingestion						
Daily ethanol consumption (g)	200.58 ± 171.49 160.00 (96.00–240.00)	-	-	179.11 ± 88.20 192.00 (125.00–240.00)	218.69 ± 218.53 140.50 (96.00–272.00)	Z = 0.38; NS
Years of addiction	32.92 ± 15.17	-	-	33.22 ± 13.17	32.66 ± 16.87	T = 0.14; NS
MCV	101.38 ± 9.25	91.97 ± 3.53	T = 6.37; *p* < 0.001	101.72 ± 12.29	101.09 ± 5.76	T = 0.26; NS
GGT	288.33 ± 348.83 175.50 (54.50–375.25)	23.06 ± 6.02 22.00 (18.50–26.00)	Z = 4.95; *p* < 0.001	405.48 ± 428.81 307.00 (76.00–611.00)	196.29 ± 220.73 92.00 (41.00–316.00)	Z = 2.25; *p* = 0.025
Albumin	3.59 ± 0.67	4.58 ± 0.32	T = 8.54; *p* < 0.001	3.57 ± 0.74	3.60 ± 0.60	T = 0.20; NS
Prothrombin	76.88 ± 19.70	97.65 ± 3.44	T = 7.70; *p* < 0.001	66.63 ± 22.1	85.53 ± 12.13	T = 3.97; *p* < 0.001
Bilirubin	2.29 ± 3.42 1.00 (1.00–2.30)	0.99 ± 0.06 1.00 (1.00–1.00)	Z = 2.73; *p* = 0.006	3.56 ± 4.72 2.00 (1.00–3.30)	1.21 ± 0.72 1.00 (1.00–1.00)	Z = 4.35; *p* < 0.001
Body composition (g) *						
Right arm fat mass	970.96 ± 529.35	-	-	1048.96 ± 526.74	889.57 ± 531.29	T = 1.03; NS
Left arm fat mass	961.00 ± 503.55	-	-	991.33 ± 459.07	929.35 ± 554.80	T = 0.42; NS
Right leg fat mass	2954.94 ±1609.91	-	-	3133.25 ± 1576.38	2768.87 ± 1658.44	T = 0.77; NS
Left leg fat mass	2929.83 ± 1609.92	-	-	3117.75 ± 1636.90	2733.74 ± 1593.53	T = 0.81; NS
Trunk fat mass	12,590.72 ± 6077.49	-	-	13,686.38 ± 6065.83	11,447.43 ± 6007.22	T = 1.27; NS
Total fat mass	20,747.47 ± 9849.06	-	-	21,936.42 ± 9699.45	19,506.83 ± 10,065.76	T = 0.84; NS
Android fat mass	2230.55 ± 1095.30	-	-	2337.71 ± 1106.44	2118.74 ± 1096.82	T = 0.68; NS
Gynoid fat mass	3489.94 ± 1929.80	-	-	3813.58 ± 2011.83	3152.22 ± 1822.60	T = 1.18; NS
Right arm lean mass	2796.26 ± 616.76	-	-	2830.46 ± 516.25	2760.56 ± 717.02	T = 0.85; NS
Left arm lean mass	2786.09 ± 663.97	-	-	2704.83 ± 568.57	2870.87 ± 754.39	T = 0.85; NS
Right leg lean mass	7460.89 ± 1796.58	-	-	7697.46 ± 1806.72	7214.04 ± 1792.05	T = 0.92; NS
Left leg lean mass	7391.47 ± 1781.77	-	-	7607.42 ± 1889.43	7166.13 ± 1673.86	T = 0.85; NS
Trunk lean mass	24,854.57 ± 4554.08	-	-	25,570.08 ± 5123.57	23,951.71 ± 3774.99	T = 1.34; NS
Total lean mass	49,044.55 ± 8623.82	-	-	50,315.71 ± 8870.36	47,718.13 ± 8345.03	T = 1.03; NS
Android lean mass	3947.15 ± 1045.88	-	-	4189.58 ± 1240.06	3694.17 ± 740.61	T = 1.65; NS
Gynoid lean mass	6276.66 ± 1571.75	-	-	6521.38 ± 1277.61	6021.30 ± 1823.58	T = 1.09; NS
Total BMC	2823.77 ± 522.96	-	-	2891.38 ± 526.60	2753.22 ± 521.29	T = 0.90; NS
Right arm BMC	250.49 ± 37.34	-	-	215.00 ± 36.71	287.52 ± 42.76	T = 0.83; NS
Left arm BMC	203.43 ± 38.35	-	-	201.25 ± 34.59	205.70 ± 42.60	T = 0.39; NS
Right leg BMC	537.30 ± 107.26	-	-	554.75 ± 111.49	519.09 ± 101.90	T = 1.14; NS
Left leg BMC	538.81 ± 111.54	-	-	549.63 ± 119.40	527.53 ± 104.15	T = 0.68; NS
Trunk BMC	870.89 ± 238.82	-	-	919.17 ± 239.15	820.53 ± 232.95	T = 1.43; NS
Total BMD	1.19 ± 0.11	-	-	1.200 ± 0.120	1.190 ± 0.110	T = 0.41; NS
Pelvis BMD	1.12 ± 0.15	-	-	1.162 ± 0.146	1.085 ± 0.147	T = 1.80; NS
Total hip BMD	0.99 ± 0.21	-	-	1.027 ± 0.197	0.949 ± 0.227	T = 1.27; NS
Spine BMD	1.12 ± 0.16	-	-	1.132 ± 0.143	1.107 ± 0.169	T = 0.54; NS

We show mean values ± SD and also the median value and interquartile range for those variables that did not show a parametric distribution. BMI: body mass index. BMC: bone mineral content. BMD: bone mineral density. * BMD is given in g/cm^3^.

**Table 2:** Relationships between myostatin and fat variables assessed by stepwise multiple regression analyses.

	Unstandardized Coefficients		Standardized Coefficients			95% Confidence Interval for B	
Variable	B	Standard Error	Beta	T	*p*	Lower Bound	Upper Bound
Serum myostatin	1.948	0.687	0.393	2.837	0.007	0.564	3.332
* Dependent variable: Fat at lower limbs. ** Non-selected variables: age, cirrhosis.							
	Unstandardized Coefficients		Standardized Coefficients			95% Confidence Interval for B	
Variable	B	Standard Error	Beta	T	*p*	Lower Bound	Upper Bound
Serum myostatin	0.004	0.002	0.324	2.270	0.028	0.000	0.007
* Dependent variable: Proportion of total fat. ** Non-selected variables: age, cirrhosis.							
	Unstandardized Coefficients		Standardized Coefficients			95% Confidence Interval for B	
Variable	B	Standard Error	Beta	T	*p*	Lower Bound	Upper Bound
Serum myostatin	0.004	0.002	0.369	2.631	0.012	0.001	0.008
* Dependent variable: Gynoid fat distribution. ** Non-selected variables: age, cirrhosis.							
	Unstandardized Coefficients		Standardized Coefficients			95% Confidence Interval for B	
Variable	B	Standard Error	Beta	T	*p*	Lower Bound	Upper Bound
Serum myostatin	0.986	0.343	0.398	2.875	0.006	0.295	1.677
* Dependent variable: Right leg fat. ** Non-selected variables: age, cirrhosis.							
	Unstandardized Coefficients		Standardized Coefficients			95% Confidence Interval for B	
Variable	B	Standard Error	Beta	T	*p*	Lower Bound	Upper Bound
Serum myostatin	0.962	0.344	0.388	2.793	0.008	0.268	1.656
* Dependent variable: Left leg fat. ** Non-selected variables: age, cirrhosis.							
	Unstandardized Coefficients		Standardized Coefficients			95% Confidence Interval for B	
Variable	B	Standard Error	Beta	T	*p*	Lower Bound	Upper Bound
Serum myostatin	2.835	1.347	0.302	2.104	0.041	0.120	5.550
* Dependent variable: Trunk fat. ** Unselected variables: age and cirrhosis.							

**Table 3:** Relationships between myostatin and BMI assessed by stepwise multiple regression analyses.

	Unstandardized Coefficients		Standardized Coefficients			95% Confidence Interval for B	
Variable	B	Standard Error	Beta	T	*p*	Lower Bound	Upper Bound
Serum myostatin	0.003	0.001	0.408	2.794	0.008	0.001	0.005

Dependent variable: body mass index. Unselected variables: age, cirrhosis.

**Table 4:** Relationships between serum myostatin and OSA criteria.

	Serum Myostatin Below the Median (705.9 pg/mL) (n = 24)	Serum Myostatin Over the Median (705.9 pg/mL) (n = 22)	T (Z); *p*
Handgrip (kp)	14.04 ± 11.51	20.23 ± 15.79	T = 1.60; *p* = 0.12
Femoral Neck T-Score	−0.64 ± 1.42	−0.62 ± 1.57	T = 0.03; NS
Appendicular lean mass/height^2^ (Kg/m^2^)	6.99 ± 1.23	7.17 ± 1.37	T = 0.44; NS
Proportion of total fat > 25% (%)	26.46 ± 7.95 27.10 (18.60–34.33)	31.32 ± 7.34 31.85 (28.05–38.33)	Z = 2.15; *p* = 0.037
Android/Gynoid fat ratio	1.00 ± 0.27	1.06 ± 0.15	T = 0.94; NS

We show mean values ± SD and also the median value and interquartile range for those variables that did not show a parametric distribution.

**Table 5:** Child–Pugh score.

Clinical and Laboratory Criteria	Points		
	1	2	3
Bilirubin (mg/dL)	<2	2–3	>3
Albumin (g/dL)	>3.5	2.8–3.5	<2.8
Prothrombin time			
%	>60	40–60	<40
International normalized ratio	<1.7	1.7–2.3	>2.3
Encephalopathy	None	Mild to moderate (grade 1 or 2)	Severe (grade 3 or 4)
Ascites	None	Mild to moderate (diuretic responsive)	Severe (diuretic refractory)

Child–Pugh Class obtained by adding score for each parameter (total points): Class A = 5 to 6 points (least severe liver disease). Class B = 7 to 9 points (moderately severe liver disease). Class C = 10 to 15 points (most severe liver disease).

## Data Availability

The data presented in this study are available on request from the corresponding author.
